# Time-dependent risk of mortality and end-stage kidney disease among patients with granulomatosis with polyangiitis

**DOI:** 10.3389/fmed.2022.817204

**Published:** 2022-08-10

**Authors:** Chun-Yu Lin, Hung-An Chen, Tsang-Wei Chang, Tsai-Ching Hsu, Chung-Yuan Hsu, Yu-Jih Su

**Affiliations:** ^1^Division of Allergy, Immunology and Rheumatology, Department of Internal Medicine, Kaohsiung Veterans General Hospital, Kaohsiung, Taiwan; ^2^Department of Health Business Administration, Fooyin University, Kaohsiung, Taiwan; ^3^Division of Allergy-Immunology-Rheumatology, Department of Internal Medicine, Chi Mei Medical Center, Tainan, Taiwan; ^4^Department of Internal Medicine, College of Medicine, National Cheng Kung University Hospital, National Cheng Kung University, Tainan, Taiwan; ^5^Institute of Biochemistry, Microbiology and Immunology, Chung Shan Medical University, Taichung, Taiwan; ^6^Department of Internal Medicine, Division of Rheumatology, Allergy and Immunology, Kaohsiung Chang Gung Memorial Hospital and Chang Gung University College of Medicine, Kaohsiung, Taiwan

**Keywords:** granulomatosis with polyangiitis, time-dependent mortality, long-term outcome, end-stage kidney disease, vasculitis

## Abstract

**Objective:**

To describe the time-dependent impact of granulomatosis with polyangiitis (GPA) on the risk of mortality and end-stage kidney disease (ESKD). The results would provide valuable insight regarding the most vulnerable period for patients with GPA.

**Methods:**

We conducted a retrospective cohort study using a nationally representative database in Taiwan. Patients with incident GPA without prior ESKD were identified, and non-GPA control cohorts were selected and matched to GPA cohorts based on sex, age, entry time and comorbidities in a 1:4 ratio. Cox regression model was used to estimate hazard ratios (HR) for mortality and ESKD stratified by the follow-up period.

**Results:**

We identified a total of 142 GPA patients and 568 matched controls. Of those, 52 GPA patients died during follow-up, 48.1% of whom did so within the first 6 months after diagnosis. The 1-, 3-, 5-, and 10-year survival rates of GPA were 78.2, 71.2, 62.6, and 54.7%, respectively. Patients with GPA exhibited the greatest risk of mortality within the first 6 months after follow-up compared with non-GPA cohorts (HR: 21.9, 95% CI: 8.41–57.5). The mortality risk diminished after 1 year and to a marginally significant level during the follow-up period of 5–10 years (HR: 2.71, 95% CI: 0.97–7.62). Ten (7.1%) of the GPA patients experienced ESKD, and these cases occurred exclusively in the first 3 years following diagnosis.

**Conclusion:**

Our findings suggest that physicians should closely monitor the treatment response and complications of patients with GPA in the first critical 6-month period after diagnosis to improve long-term survival outcome.

## Introduction

Granulomatosis with polyangiitis (GPA) is a form of primary systemic vasculitides of unknown etiology with significant rates of morbidity and mortality (1, 2). The condition is characterized by granulomatous and necrotizing inflammation that predominantly affects small-sized to medium-sized blood vessels (3). The upper respiratory tract, lungs, and kidneys are the preferentially affected organs of GPA, and lethal complications, such as pulmonary hemorrhage or uremia, may occur (2, 4). The presence of circulated anti-neutrophil cytoplasmic antibodies (ANCAs) in the serum in the majority of patients with GPA is considered the immunological hallmark (5). Recently, some evidence has suggested potential geographic variations in the clinical features of GPA. For example, up to 60% of Japanese and Chinese patients with GPA had anti-myeloperoxidase autoantibodies (6, 7). In contrast, the predominant ANCA subtype in patients from European countries was anti-proteinase 3 antibodies (1). Still, whether the long-term outcomes and risks of end-stage kidney disease (ESKD) also differ between Western and Asian countries remains unknown. As the incidence of GPA is much higher in Western countries and a rarity in Asian countries, the majority of published epidemiological studies have been carried out in Europe and North America (8).

Although a dramatic improvement of prognosis followed the introduction of intense immunosuppression with pulse cyclophosphamide and corticosteroids regimen in recent decades, long-term survival in patients with GPA has remained a major concern (9). However, previous studies of GPA survival in such Asian countries as Japan (7) or Korea (10) have been limited by small sample size, usually less than 50 patients, were lacking control groups, and were single center study designs, which may introduce sampling and/or referral bias. Furthermore, studies assessing the separate mortality risk of GPA based on the different time interval during follow-up were scarce.

Therefore, we aimed to conduct an epidemiological study to describe the long-term patient survival and renal outcome of GPA in an unselected cohort using a nationwide database in Taiwan. We further sought to estimate the risk of death according to different follow-up periods after GPA diagnosis, which could provide insight with regard to the clinical course of GPA and risk stratification strategies to manage GPA patients.

## Materials and methods

### Study design, data source, and ethical approval

This study was carried out using a retrospective study design. Data were retrieved from the Taiwan National Health Insurance Research Database (NHIRD).^1^ A compulsory single-payer social health insurance program was launched in Taiwan in 1995. Approximately 99% of citizens in Taiwan are enrolled in this insurance program, which covers a broad range of medical services, including ambulatory care, inpatient care, emergency care, and various medications and surgical procedures. NHIRD was constructed from the claims data of beneficiaries enrolled in this national program, making it one of the most comprehensive databases in the world. NHIRD has thus provided an enriched source for conducting epidemiological studies and generating real-world evidence (11, 12). Informed consent was waived due to the retrospective nature of the study and de-identified data from NHIRD. The study was approved by the institutional review board of National Cheng Kung University Hospital (A-EX-109-017).

### Study population

Adult patients (aged ≥ 18 years) who were diagnosed with GPA for the first time were captured by the International Classification of Diseases, Ninth Revision, Clinical Modification (ICD-9-CM) diagnostic code 446.4 during the period of January 1, 2000 to December 31, 2013. To ensure the accurate identification of GPA patients, we also examined the catastrophic illness registry, a subset database of NHIRD. In Taiwan, patients with serious diseases, such as cancers, ESKD, and various autoimmune diseases, can apply to be enrolled in the catastrophic illness registry and then exempted from co-payment when seeking medical services related to their corresponding illness. Two independent medical experts are required to review the medical records, laboratory data, and radiographic images prior to approving each individual applicant with a specific catastrophic illness. Therefore, in order to be issued a catastrophic illness certificate for GPA, the 1990 American College of Rheumatology criteria for GPA (13) should be fulfilled, or the relevant symptoms/sign were highly compatible with GPA and cannot be explained by other medical conditions. The aforementioned certification process will be reviewed by at least two independent board certified rheumatologists to ensure the correctness. If patients received a diagnostic code for GPA but were not included in the catastrophic illness registry, we did not consider them as having GPA and excluded them from the current study. The identification of patients with GPA using this selection process was highly valid. The index date was defined as the date of diagnosis of GPA, and we also searched whether a diagnosis of GPA had been made in the preceding 2 years prior to the index date to ensure that only incident cases were included in the GPA cohort. GPA patients also diagnosed with other autoimmune diseases were excluded to ensure that we did not capture overlap syndrome in our study.

### Comparison cohort and covariate assessment

We assembled a general population comparison cohort by identifying participants who had never been diagnosed with GPA during the study period. The index date of the non-GPA cohort was randomly assigned, matching to the index date of the GPA cohort. Six types of comorbidities were assessed in the baseline period (within 1 year before the index date). These comorbidities were defined according to their corresponding ICD-9-CM codes, as well as by whether the given diagnosis was recorded at least 3 times in outpatient visit claims or at least once in an inpatient visit claim. The identified comorbid conditions are listed as follows: diabetes mellitus (ICD-9-CM code 250), hypertension (ICD-9-CM codes 401-405), chronic kidney disease (ICD-9-CM codes 580-588), ischemic heart disease (ICD-9-CM codes 410-414), stroke (ICD-9-CM codes 433-434), and hyperlipidemia (ICD-9-CM code 272). An exact matching approach was adopted in the current study to eliminate the confounding effect of covariates. That is, each patient with GPA was individually matched to four control subjects with regard to age, sex, and medical chronic comorbidities.

### Utilization of therapeutic agents for granulomatosis with polyangiitis

Various immunosuppressants used to treat GPA were assessed after the index date. The drugs included in this study consisted of intravenous and oral cyclophosphamide, azathioprine, hydroxychloroquine, methotrexate, sulfasalazine, mycophenolate mofetil, cyclosporine, and leflunomide. Use of certain immunosuppressive agents was defined by at least one dispensation over 7 days at any point during the follow-up period.

### Follow-up and determination of outcome

The primary outcome measure was all-cause mortality. The secondary outcome was development of ESKD. The development of ESKD was determined by the catastrophic illness certificate. As described above, ESKD is considered one of the catastrophic illnesses in Taiwan, and certified nephrologists must approve the application for catastrophic illness certificate due to ESKD. Patients in the GPA or comparison cohorts who experienced ESKD before the index date were excluded. All participants were followed from the index date until the occurrence of death or the study’s end date (December 31, 2013), whichever came first.

### Statistical analysis

Continuous variables were presented as mean ± standard deviation, and categorical variables were expressed as number and percentage. Student’s *t*-test or chi-square test were used to determine the difference of variables between GPA and comparator groups, as appropriate. Incidence rates (IRs) and the incidence rate ratio of mortality and ESKD per 100 person-years were estimated using Poisson regression. Long-term survival and the cumulative incidence of ESKD were calculated and expressed graphically using the Kaplan–Meier method. We adopted the log-rank test to compare and determine whether a significant difference of risk for mortality and ESKD existed between the GPA and non-GPA cohorts. Meanwhile, we used Cox proportional hazard regression to generate hazard ratios and 95% confidence intervals for the primary and secondary outcome measures. The Cox analysis for risk of mortality was further stratified according to time since start of follow-up and sex to assess the impact of these two variables on the outcome. When estimating the risk of ESKD, we further carried out competing risk analysis using the Fine and Gray model to account for the high mortality rate in patients with GPA (14). All data management and statistical analyses were performed using the statistical package, STATA, version 13.0 (StataCorp, College Station, Texas, United States). The statistical tests were two-sided, and *P* < 0.05 was considered statistically significant.

## Results

### Demographics and baseline characteristics

Descriptive statistics and comorbidities at baseline of the GPA cohorts and matched general population are presented in Table 1. After excluding two GPA patients who developed ESKD prior to the index date, a total of 142 adult GPA patients and 568 non-GPA controls were identified during the study period. The mean age at the diagnosis of GPA was 52.6 years, and the percentage of male sex was slightly higher than their female counterparts. The most common baseline comorbid condition was hypertension, followed in order by diabetes, chronic kidney disease, ischemic heart disease, and stroke. The mean duration of follow-up of all patients was 5.1 years.

**TABLE 1:** Baseline demographics and comorbidities in patients with granulomatosis with polyangiitis (GPA) and control cohorts.

Characteristics	GPA (*n* = 142)	Non-GPA (*n* = 568)	*P*-value
**Sex, *n* (%)**			
Male	73 (51.4)	276 (51.4)	0.99
Female	69 (48.6)	292 (48.6)	
**Age**, years, mean ± SD	52.6 + –14.3	52.8 + –13.8	0.89
**Comorbidities, *n* (%)**			
Diabetes mellitus	23 (16.2)	92 (16.2)	0.99
Hypertension	35 (24.6)	140 (24.6)	0.99
Chronic kidney disease	19 (13.4)	76 (13.4)	0.99
Ischemic heart disease	7 (4.9)	28 (4.9)	0.99
Stroke	3 (2.1)	12 (2.1)	0.99
Hyperlipidemia	15 (10.6)	60 (10.6)	0.99

### Prescribing patterns of immunosuppressants among granulomatosis with polyangiitis patients

Table 2 shows the percentage of real-world utilization of various systemic immunosuppressive drugs in our GPA cohorts. Not surprisingly, cyclophosphamide was the most commonly prescribed medication for the treatment of GPA, and over 80% of patients received an oral or intravenous form of this potent agent at some point. Approximately half of patients had used azathioprine during the course of GPA, which is the second most commonly used immunosuppressive drug. Hydroxychloroquine and methotrexate were administered to 44 (31.0%) and 37 (26.1%) GPA patients, respectively. Sulfasalazine, mycophenolate mofetil, cyclosporin, and leflunomide were rarely used to treat GPA, all less than 10%.

**TABLE 2:** Number and percentage of prescribed immunosuppressants among the 142 patients with granulomatosis with polyangiitis.

Immunosuppressants/ Immunomodulators	Number of patients (%)
Cyclophosphamide (oral or intravenous)	118 (83.1)
Azathioprine	68 (47.9)
Hydroxychloroquine	44 (31.0)
Methotrexate	37 (26.1)
Sulfasalazine	11 (7.8)
Mycophenolate mofetil	6 (4.2)
Cyclosporin	5 (3.5)
Leflunomide	2 (1.4)

### Risk of mortality and end-stage kidney disease

**FIGURE 1:**
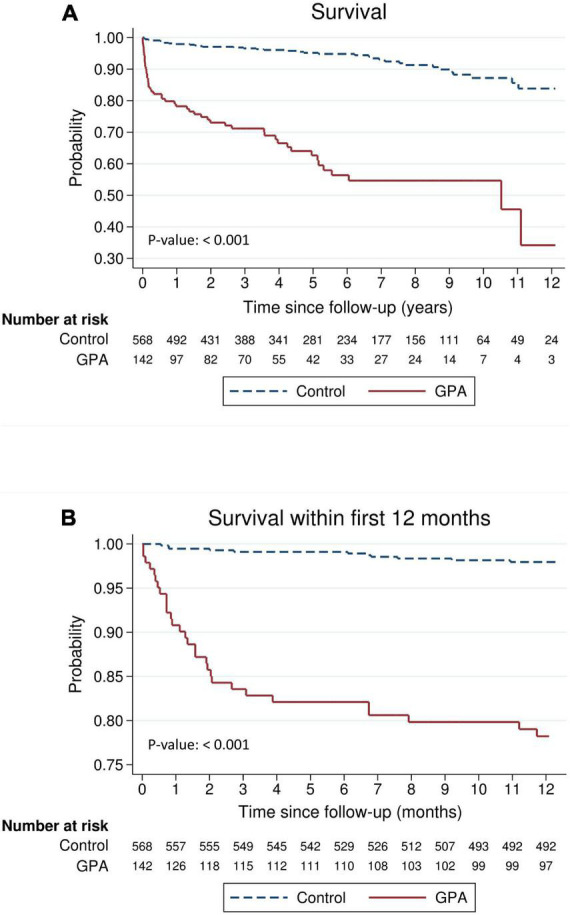
Survival of patients with granulomatosis with polyangiitis (GPA) compared with an age-, sex-, entry time- and comorbidity-matched general population **(A)** during the entire follow-up period or **(B)** within the first 12 months.

**FIGURE 2:**
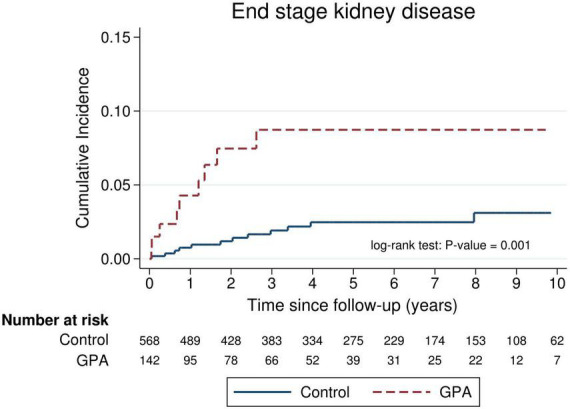
Cumulative incidence of end-stage kidney disease (ESKD) in incident patients with granulomatosis with polyangiitis (GPA) compared with an age-, sex-, entry time- and comorbidity-matched control.

The absolute and relative risks of the outcomes of interest are presented in Table 3. Death was observed in 52 (36.6%) and 37 (6.5%) of the GPA patients and matched controls, respectively, which corresponded to an incidence rate of 10.0 and 1.2 per 100 person-years, respectively. The incidence rate ratio of mortality in GPA vs. controls was 8.23 (95% CI: 5.30–12.9). Figure 1 shows long-term survival probability and the first 12 months’ survival rate. Survival of patients with GPA was significantly lower compared with their matched controls (log-rank test, *P*-value < 0.001) (Figure 1A). Regarding the timing of mortality, a steep descent of survival rate occurred around the first 6–12 months after the diagnosis of GPA (Figure 1B). Among the total 52 deaths, 48.1% occurred within the first 6 months, and 57.7% occurred within 1 year following the GPA diagnosis. The 6-month survival rate was 82.1% and the 1-, 3-, 5-, and 10-year survival rates in GPA patients were 78.2, 71.2, 62.6, and 54.7%, respectively. The Cox model revealed that the risk of death was seven times greater in the GPA cohort than those in the matched general population (HR: 7.74, 95% CI: 5.06–11.83) (Table 3). Ten (7.1%) and 12 (2.1%) patients experienced ESKD among the GPA and matched control groups, respectively. The incidence rates of ESKD were estimated to be 2.0 per 100 person-years for GPA cohorts and 0.4 per 100 person-years for the compared general population. ESKD occurred exclusively in the first 3 years following the GPA diagnosis (Figure 2). Cox regression analysis revealed that the risk of ESKD in patients with GPA was 4.4 times higher than that of the matched control groups (HR: 4.44, 95% CI: 1.91–10.31). The sub-distribution hazard ratio (sHR) derived from the competing risk regression showed a similar strength and direction of risk of ESKD (HR: 3.42, 95% CI: 1.48–7.92) as the Cox model.

**TABLE 3:** Incidence rate (per 100 person-years) and hazard ratio of mortality and end-stage kidney disease (ESKD) in patients with granulomatosis with polyangiitis (GPA) compared with the non-GPA population.

	GPA (*n* = 142)	Non-GPA (*n* = 568)	*P*-value
**Mortality**			
Cases, *n* (%)	52 (36.6)	37 (6.5)	
Person-years	520.3	3044.9	
Incidence rate (95% CI)	10.0 (7.5–13.1)	1.2 (0.9–1.7)	
Incidence rate ratio (95% CI)	8.23 (5.30–12.9)	1 (reference)	<0.001
Hazard ratio (95% CI)	7.74 (5.06–11.83)	1 (reference)	<0.001
**ESKD**			
Cases, n (%)	10 (7.1)	12 (2.1)	
Person-years	494.6	3003.4	
Incidence rate (95% CI)	2.0 (0.9–3.7)	0.4 (0.2–0.7)	
Incidence rate ratio (95% CI)	5.06 (1.96–12.78)	1 (reference)	<0.001
Hazard ratio (95% CI)	4.44 (1.91–10.31)	1 (reference)	0.001
Subdistribution hazard ratio (95% CI)*	3.42 (1.48–7.92)	1 (reference)	0.004

*Calculated by competing risk regression model.

### Mortality risk stratified by follow-up duration and sex

Stratified analysis of mortality risk by the duration of follow-up and sex is shown in Table 4. The highest risk of mortality was observed in patients with GPA within the period of the first 6–12 months after the index date compared with non-GPA cohorts (HR: 21.9, 95% CI: 8.41–57.5 at 6 months; HR: 12.4, 95% CI: 6.19–24.7 at 12 months). The magnitude of mortality risk decreased after 1 year following the GPA diagnosis and attenuated to a marginally significant level during the follow-up period of 5–10 years (HR: 2.71, 95% CI: 0.97–7.62). Separate analyses according to male and female sex revealed a similar trend.

**TABLE 4:** Risk of death in patients with granulomatosis with polyangiitis (GPA) stratified by the time frame since the start of follow-up and by sex.

	Total		Male		Female	
Time after diagnosis	GPA (*n* = 142)	Non-GPA (*n* = 568)	GPA (*n* = 73)	Non-GPA (*n* = 292)	GPA (*n* = 69)	Non-GPA (*n* = 276)
**0–6 months**						
No. of death, n (%)	25 (17.7)	5 (0.9)	15 (20.5)	5 (1.7)	10 (14.5)	0
HR for death (95% CI)	21.9 (8.41–57.5)	1.0 (reference)	13.3 (4.82–36.5)	1.0 (reference)	–	1.0 (reference)
*P*-value	<0.001		<0.001		–	
**0–12 months**						
No. of death, *n* (%)	30 (21.1)	11 (1.9)	18 (24.7)	9 (3.1)	12 (17.4)	2 (0.7)
HR for death (95% CI)	12.4 (6.19–24.7)	1.0 (reference)	9.07 (4.07–20.2)	1.0 (reference)	27.0 (6.1–120.7)	1.0 (reference)
*P*-value	<0.001		<0.001		<0.001	
**1–5 years**						
No. of death, *n* (%)	15 (10.6)	11 (1.9)	8 (11.0)	4 (1.4)	7 (10.1)	7 (2.5)
HR for death 95% CI)	7.65 (3.51–16.7)	1.0 (reference)	10.9 (3.28–36.2)	1.0 (reference)	5.76 (2.02–16.5)	1.0 (reference)
*P*-value	<0.001		<0.001		0.001	
**5–10 years**						
No. of death, *n* (%)	5 (3.5)	13 (2.3)	1 (1.4)	5 (1.7)	4 (5.8)	8 (2.9)
HR for death (95% CI)	2.71 (0.97–7.62)	1.0 (reference)	1.25 (0.15–10.7)	1.0 (reference)	3.96 (1.18–13.2)	1.0 (reference)
*P*-value	0.058		0.83		0.025	

### Mortality risk by early (2000–2009) and late (2010–2013) granulomatosis with polyangiitis cohort

We divided the patients with GPA into two subgroups based on the year of diagnosis: early GPA cohort (2000–2009) and late (2010–2013) GPA cohort. A total of 55 GPA patients were identified in the early cohort and 87 GPA patients were identified in the late cohort (Table 5). Cox model showed that the risk of death tended to be lower in late GPA cohort (2010–2013) compared with that in early GPA cohort (2000–2009) (HR: 0.67, 95% CI: 0.32–1.40, *P*-value: 0.19), but the difference did not reach a significant level.

**TABLE 5:** Hazard ratio (HR) for mortality in patients with granulomatosis with polyangiitis (GPA) according to calendar time-based subgroups.

	Early GPA cohort (2000–2009)	Late GPA cohort (2010–2013)
Patient numbers	55	87
HR for mortality	1 (reference)	0.67
Confidence interval of HR	–	0.32–1.40

## Discussion

To the best of our knowledge, this study is the first to investigate the differential risk of mortality and ESKD according to follow-up duration using a nationally representative database in Taiwan. Our results have indicated that patients with GPA exhibit a much higher risk of mortality in the first year after the start of follow-up, particularly in the first 6 months, compared to the general comparison cohorts. However, the risk of mortality decreased thereafter, diminishing to an insignificant level after 5 years’ follow-up. The development of ESKD in patients with GPA occurred exclusively in the first 3 years following the disease diagnosis.

We observed that the excess mortality of GPA was extremely high within the first year, particularly the first 6 months after diagnosis, compared to the matched comparison cohort. Previously published clinic-epidemiologic studies regarding GPA in Japan, Korea, and other Asian countries did not report timing of death or mortality risk stratified by follow-up duration due to limited sample size or insufficient tracking time (7, 10, 15). A pooled analysis from randomized clinical trials documented that the causes of death in GPA differ in patients with early death compared to those with late death (9). Infection accounted for 48% of death within the first year after GPA diagnosis, followed by active vasculitis (19%). In the group of late death (> 1 year), cardiovascular disease (26%), malignant tumors (22%), and infection (20%) are the most common etiologies of mortality (9). Earlier studies by Mahr et al. showed that the majority of infectious complications developed within the first 6 months following diagnosis (16). Taken together, it is reasonable to speculate that the main cause of early death in our cohort could be attributed to infection. Our results emphasize the critical importance of proper management in the initial period of presentation of GPA, as well as of seeking a balance between achieving timely control of disease activity and lowering infection risk due to intense immunosuppression.

Despite treatment advances that have been made for decades, GPA is still associated with a high mortality rate (8, 17). Earlier studies revealed a considerable variation, with the majority coming from European countries, which assessed the long-term survival rate of GPA and reported a survival rate of from 85 to 97% at 1 year, 69 to 91% at 5 years, and 75 to 88% at 10 years (18). This variation may be explained by the variable number of enrolled patients, differing disease severity, and hospital-based or population-based cohort. A more recent study from the French Vasculitis Study Group revealed a 10-year survival rate of up to 88.2% (19). Another multicenter study that pooled GPA patients from four randomized trials also showed a comparable result regarding survival (9). However, our cohorts, which are representative of the Taiwanese population, indicate a worse long-term prognosis compared with that in Western countries. This observation may be partially explained by ethnicity, different distribution patterns of ANCA seropositivity (5–7, 20–22), and the use of a single hospital-based or general practice database in European countries vs. the use of a nationwide database in the current study. Another possible explanation for the differing outcome may have to do with the algorithm in this study that were used to capture patients with GPA. Although our algorithm to identify GPA was based on the information regarding catastrophic illness certificate with expected high validity and specificity, there was chance that a small fraction of GPA patients with very mild symptoms/signs or limited disease were not identified in our cohort. This may lead to higher morality and poorer prognosis observed in our results. With respect to the treatment regimen, the most frequently prescribed maintenance therapies were cyclophosphamide (93.6%), azathioprine (48.1%), methotrexate (25.9%), or mycophenolate mofetil (7.4%) according to a registry study from France (19). This prescribing pattern was comparable to our results. However, there were insufficient data regarding the dosage of immunosuppressants used for GPA, which preclude further assessment of the impact of treatment strategy on the different survival rate between European countries and Taiwan.

As we did not gain access to the cause of death in our study, exploring whether the Han Chinese population is more susceptible to infectious complications under the current recommended dosage of immunosuppressants or whether the clinical expression of GPA and the cumulative organ damage resulting from the disease itself differed between Chinese and Caucasian populations was not possible. Nevertheless, the comparative results of the overall all-cause mortality pattern are of paramount importance in itself.

A recent single-center study by Shi et al. which analyzed the outcome of 124 Chinese patients with microscopic polyangiitis (MPA) revealed that 6-month and 1-year survival rates were 83.9 and 78.2%, respectively (23). Shi et al. also showed that the peak mortality of their MPA cohort was within the first 6 months after diagnosis. Although the survival rate and mortality pattern of Chinese MPA cohort was highly comparable to our patients with GPA, it should be noted that all MPA patients enrolled in the Shi’s study had varying degree of renal involvement, making it difficult to make a comparison of prognosis directly between GPA and MPA in this context.

The present study demonstrated that all of patients with GPA progressed to ESKD in the first 3 years of their disease. A recent large-scale epidemiologic study from US using a national claims database revealed that 319 (17%) of 1,875 incident GPA cases developed ESKD (24), and this percentage was higher than our results. With regard to the timing of irreversible renal damage, this investigation observed that among patients who developed ESKD, 89.9% of them did so in the first year following the GPA diagnosis (24). This pattern of early ESKD occurrence in the course of GPA was comparable to our findings.

Our data suggested that there was a trend of improved survival of GPA over past decades. This observation may be attributed to better and earlier GPA recognition by physicians, increased availability of ANCA testing, improved management of therapy-related complications. As we did not estimate the cumulative dosage of glucocorticoids in our study, the full impact of glucocorticoids on the survival benefit was unknown. Further studies will be needed to elucidate the exact reason behind this observation.

The current study has some particular strengths, including the use of a nationwide population-based database with near complete coverage to establish an unselected GPA cohort, which substantially reduced selection bias. We also used a highly validated algorithm to correctly identify patients being diagnosed with GPA and determine the outcomes of interest. Therefore, misclassification bias, which was usually a non-negligible concern in observational epidemiological studies, could be minimized. We also conducted competing risk regression analysis to account for the high mortality rate of GPA in order to evaluate the robustness of our results.

However, certain limitations of this study also ought to be mentioned. First, although the use of a national database enabled us to enroll all GPA patients in Taiwan, only 142 patients were ultimately identified during the 14-year period due to the low incidence of GPA in Asian countries, which precludes further subgroup analysis to assess interaction effects between mortality and various covariates. Second, detailed associated symptoms/signs of GPA and some laboratory measures, such as levels of ANCA, were not available in our database. Therefore, we were unable to evaluate disease activity and could not further designate patients with GPA into groups of varying severity. However, whether serum levels of ANCA in GPA are reflective of disease activity or not remains uncertain. We believe that the lack of data regarding ANCA would not influence our results. Third, we did not analyze the impact of various immunosuppressive drugs on the survival and risk of ESKD, although we did evaluate the utilization pattern of these drugs in the course of GPA. As disease activity could not be evaluated in our GPA population as mentioned above, including the binarized measures for use of immunosuppressants in the regression model would not prudent and may have led to protopathic bias since drugs with more intense immunosuppressive properties would be administered to patients with higher disease activity who inherently carried greater risk of complications and mortality. Fourth, owing to lack of information regarding the exact cause of mortality in patients with GPA, it is difficult to attribute the extremely high mortality rate in the first 6–12 months after diagnosis to the high activity and severity of the GPA itself, or infectious complications, or malignancy, or cardiovascular diseases. This is a great limitation of this study and it should be cautions when interpreting our results. Fifth, the type and frequency of extrarenal manifestations of GPA, such as lung involvement, were not examined in our study. Hence, we are unable to further analyze the differential impact of each major organ involvement on the risk of death. Sixth, while rituximab, a chimeric monoclonal antibody that binds specifically to the CD20 antigen, was an effective therapy for the induction and maintenance of remission in GPA, it has not been reimbursed for the treatment of GPA in Taiwan’s national health insurance system until September 1, 2014. Therefore, the influence of rituximab on the mortality of GPA cannot be assessed in our study and it should be noted that high risk of death in GPA within the first 12 months after its diagnosis may improve over the past decade due to applied use of rituximab in GPA. Seventh, as the pulmonary manifestations of GPA, including pulmonary nodules, ground glass opacities, or patches of consolidation, were not commonly seen in the non-GPA cases or otherwise healthy individuals, it may be more appropriate to choose patients with MPA as the control group in this study from this point of view. However, the algorithm of capturing MPA cases and its validity have not been established in our national database. Thus, we are unable to compare the outcome and mortality pattern of GPA with MPA directly in our database.

In conclusion, our study indicates that patients exhibit a much higher risk of mortality within the first 6–12 months following the diagnosis of GPA and that said risk decreased after 5 years’ follow-up to a similar level to the age-, sex-, entry time- and comorbidity matched individuals. Progression to ESKD also occurs in the first few years in the course of GPA. These findings call for awareness and increased vigilance of the critical initial treatment phase of GPA and highlight the importance of striking a balance between gaining efficacy of the therapeutic regimen while preventing treatment-related complications.

## Data availability statement

The original contributions presented in the study are included in the article/supplementary material, further inquiries can be directed to the corresponding author/s.

## Ethics statement

The studies involving human participants were reviewed and approved by the National Cheng Kung University Hospital. Written informed consent for participation was not required for this study in accordance with the national legislation and the institutional requirements.

## Author contributions

Y-JS: full access to all of the data in the study and take responsibility for the integrity of the data and the accuracy of the data analysis. C-YL: concept and design. C-YL, T-WC, and Y-JS: acquisition, analysis, and interpretation of data. C-YL and T-WC: drafting of the manuscript and statistical analysis. C-YL and Y-JS: administrative, technical, and material support, and supervision. All authors: critical revision of the manuscript for important intellectual content.

## Footnotes

1
http://nhird.nhri.org.tw/en/index.html
